# Thoracic empyema due to nontuberculous mycobacteria in an immunocompetent patient without pulmonary disease: a case report

**DOI:** 10.1186/s12890-023-02494-5

**Published:** 2023-06-19

**Authors:** Fengjiao Yu, Yongxia Li, Jing Luo, Xingru Chen, Yu Jiang

**Affiliations:** Department of Respiratory and Critical Care Medicine, University-Town Hospital of Chongqing Medical University, Chongqing, 401331 People’s Republic of China

**Keywords:** Diagnostic dilemma, Immunocompetent, Empyma, MAC, mNGS, Histopathology

## Abstract

**Background:**

Pleural involvement by non-tuberculous mycobacteria (NTM), especially NTM empyema in the immunocompetent patient without pulmonary diseases is a rare disease. It is difficult to diagnose with only a few cases of immunodeficient patients in the literature.

**Case presentation:**

We describe a 63-year-old male with empyema due to NTM and highlight the challenges of diagnosis.

**Conclusions:**

Non-tuberculous mycobacterial infection should be considered as a cause of pleuritis or empyema without pulmonary disease, however it is a real diagnostic dilemma.

## Background

Non-tuberculous mycobacteria (NTM), are well-known as a pathogen of lungs, lymphadenitis, soft tissues, and bone [1, 2]. NTM, especially M. avium-intracellulare complex (MAC) [2, 3], occasionally causes pleurisy or empyema, probably due to direct spread from pulmonary lesions [4].

However, empyema without distinct pulmonary disease in the immunocompetent patient is rarely reported. On the one hand, it is a so rare clinical presentation of NTM infection that we ignore it. On the other hand, it is very difficult to differentiate nontuberculous empyema from a tuberculous infection. There is a lack of modern facilities for diagnosis, especially in resource poor areas and countries.

Herein, we report a case of empyema without distinct pulmonary disease caused by MAC. In addition, the literature is reviewed and the present diagnostic methods of NTM empyema are summarized to improve the diagnosis of this dilemma worldwide.

## Case presentation

The patient, a 63-year-old man, was admitted to our hospital in July 2021 due to a cough lasting for over 10 days. Two days before the admission, the cough got worse and was accompanied by dyspnea and fever with a body temperature of 38.3℃. There were no other systemic symptoms.

The patient was an alcoholic and a never-smoker. He had suffered from hypertension for over 10 years with stable blood pressure. He had no history of chronic lung disease or diabetes and had no contact to tuberculosis patients.

A physical examination revealed he was acutely ill-looking. He was febrile (38.9℃), and in moderate respiratory distress severely with a respiratory rate of 28 cycles/min. He had tachycardia of 130 beats/min. There was markedly reduced breath sound intensity on the right hemithorax. On the auscultation of both lungs, the breath sounds were decreased at the right lung base. No positive signs were found in the examinations of the other systems.

**Fig. 1:**
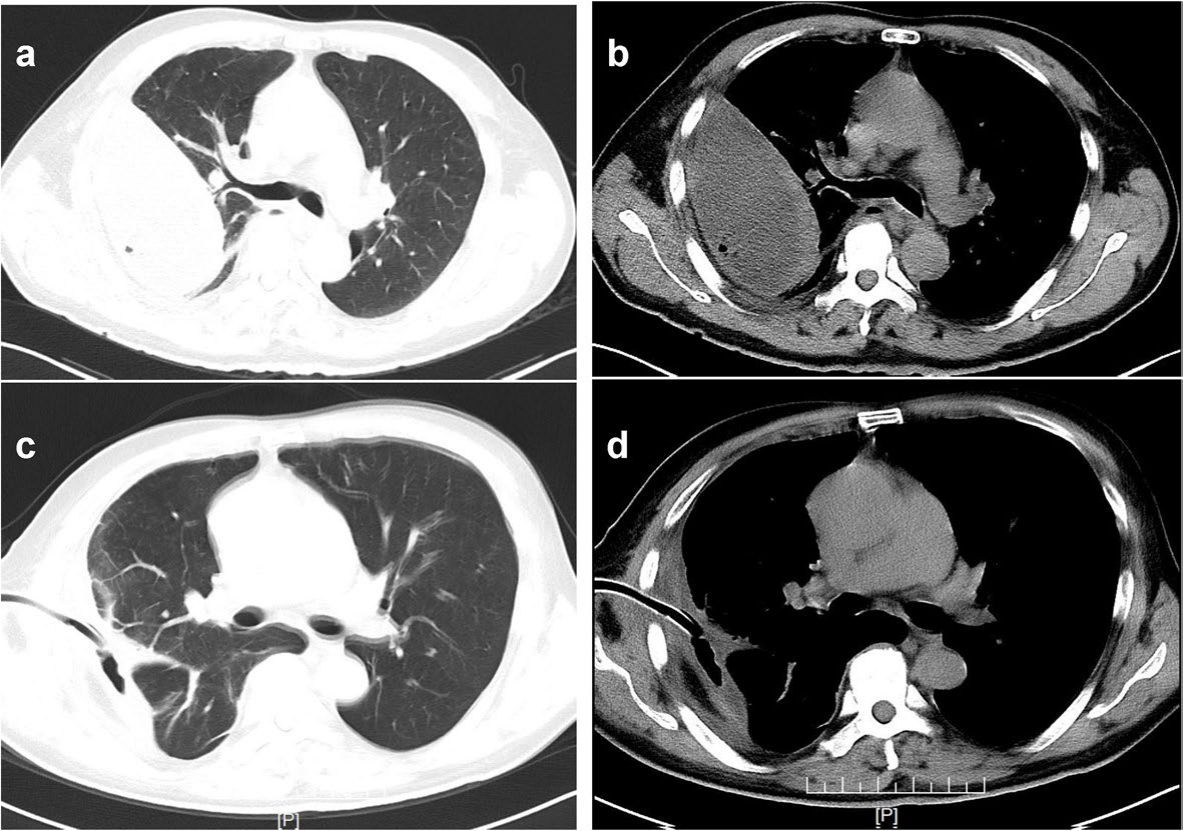
Chest computed tomography (CT) obtained on admission (**a**) chest window e (**b**) mediastinal window showing located right pleural effusion. Chest CT performed at two weeks after the start of antimicrobial treatment (**c**) chest window e (**d**) mediastinal window demonstrated a decrease in right pleural effusion and thickening of the right pleura

The initial laboratory investigations (Table 1) revealed the following results: Full blood count revealed a white blood count of 18.33 × 10^9^/L; neutrophil count of 87.4%; hemoglobin of 155 g/L; and an elevated C-reactive protein level (CRP) (257.10 mg/L) and procalcitonin(PCT) concentration (14.90 ng/mL). The test results for tumor markers, human immunodeficiency virus, a routine sputum smear, Ziehl-Nielsen staining, and cultures (included Fungal,not Mycobacterium culture) were all negative. Chest computed tomography (CT) on admission also demonstrated right pleural effusion with no abnormal shadows in the bilateral lung fields (Fig. 1).

**Table 1:** The results of the peripheral blood analysis on admission

< Blood cell counts >	< Blood chemistry >	< Infection >
WBC 18.33*10^9 /L	TP 67.30 g/L	Sputum TB-DNA (**-**)
Neut 87.74%	Alb 36.60 g/L	Bacterial culture (-)
Lymph 6.2%	ALT 14.80 U/L	IGRA (-)
EOS 0.0%	AST 21.50 U/L	
RBC 4.93*10^12 /L	LDH 121U/L	
HB 155 g/L	ALP 100 U/L	
PLT 318*10^9 /L	BUN 6.91 mmol/L	HIV antibody (-)
	Cre 89.80 μmol/L	
D-dimer 4.27 mg/L	Na 132.80 mmol/L	
	K 3.16 mmol/L	

Bacterial cultures(included Fungal,not Mycobacterium culture)

*WBC* White blood cell, *Neut* Neutrophilic granulocyte, *Lymph* Lymphocyte, *EOS* Eosinophil, *RBC* Red blood cell, *HB* Hemoglobin, *PLT* Platelet, *TP* Total protein, *Alb* Albumin, *ALT* Alanine-aminotransferase, *AST* Aspartate aminotransferase, *LDH* Lactatec dehydrogenase, *ALP* Alkaline phospaatase, *BUN* Blood urea nitrogen, *Cre* Creatinine, *Na* Sodium, *K* Kalium, *HIV* Human immunodeficiency virus, *TB-DNA* Tuberculous-deoxyribonucleic acid, *IGRA* Interferon-gamma release assay

The right pleural effusion was yellow initially. Laboratory analysis of the pleural effusion (Table 2) demonstrated neutrophil predominance(97%). The pleural fluid had a protein concentration of 56.71 g/L, an adenosine deaminase(ADA) concentration of 36.67U/L, and a marked increase in the lactic acid dehydrogenase level (LDH) of 2096.13U/L. In addition, no bacteria (include Mycobacterium culture) were detected in the pleural effusion bacterial cultures. Ziehl–Neelsen staining revealed no acid-fast bacilli and the Mantoux test was nonreactive. Meanwhile, polymerase chain reaction(PCR) in house was used to check and measure the tubercle bacillus in the pleural fluid and sputum samples were negative.

**Table 2:** The results of the pleural effusion laboratory analysis

< on admission >	< 10 days after admission >	< 23 days after Admission >	< 14 days after treatment gainst MAC >
Color Yellow	Yellow	Purulent yellow	Yellow
Cell count, 3794010^6 /L	15,299	33,000	87,530
WBC, 23,250 10^6 /L	11,299	23,250	48,530
Lymph, % 3	8	8	10
Neut, % 97	92	92	90
TP, g/L 56.71	47.80	51.73	41.90
Alb, g/L 30.54	25.47	24.02	17.50
Tbil, μmol/L 17.93	15.25	10.14	11.53
LDH, U/L 2096.13	5883.38	7576.41	4757.30
ADA, U/L 36.67	81.87	118.82	86.22

Cefuroxime (3.0 g/day) was initiated after admission to treat right pleurisy and the chest tube underwent drainage but it did not completely stop the fever. One week later, it changed to Imipenem (2.0 g/day)/Cilastatin Sodium (2.0 g/day) for ten days and his temperature is normal after 3 days of this antibiotics therapy. Ten days after his admission, the laboratory findings demonstrated a normal white blood cell count(7.38 × 10^9^/L) and the CRP decreased (63.5 mg/dL). But an analysis of the pleural effusion revealed that the ADA level was increased (83.87 U/L) (Table 2). Although the IGRA for M. tuberculosis was negative and the neutrophil predominance of the right pleural effusion, the elevated ADA level of pleural effusion was suggestive of tuberculous pleurisy. Given the clinical and laboratory findings, isoniazid (INH) (300 mg/day), rifampicin (RFP) (450 mg/day), ethambutol (EB) (750 mg/day), and moxifloxacin (400 mg/day), a diagnostic trial therapy, were started on the ninth day after admission.

**Fig. 2:**
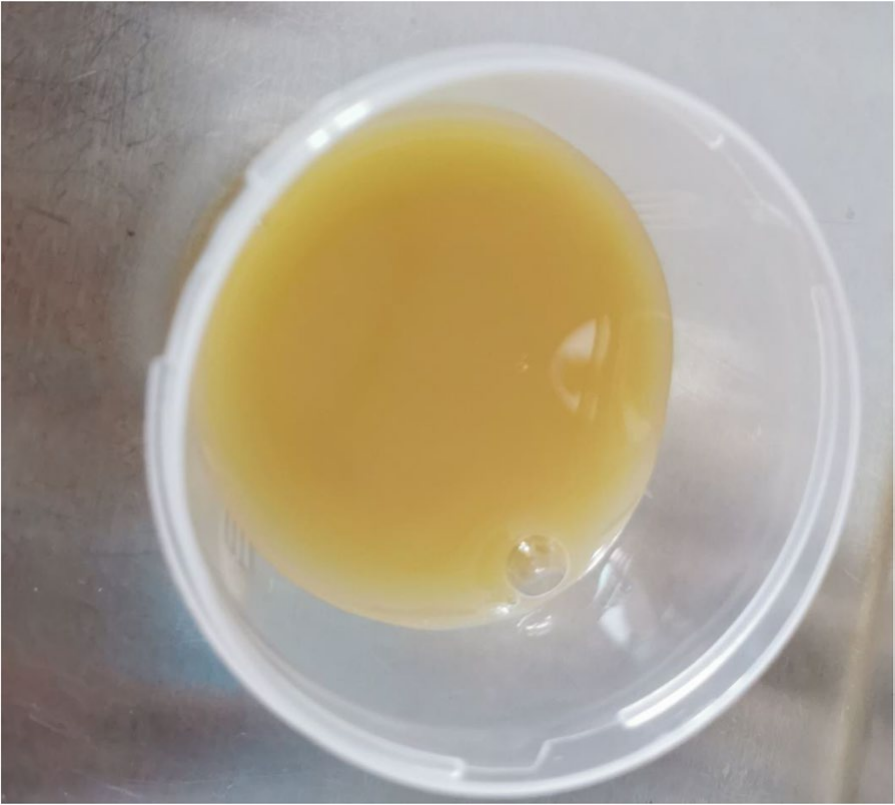
A purulent yellow color of the pleural effusion 23 days after his admission

**Fig. 3:**
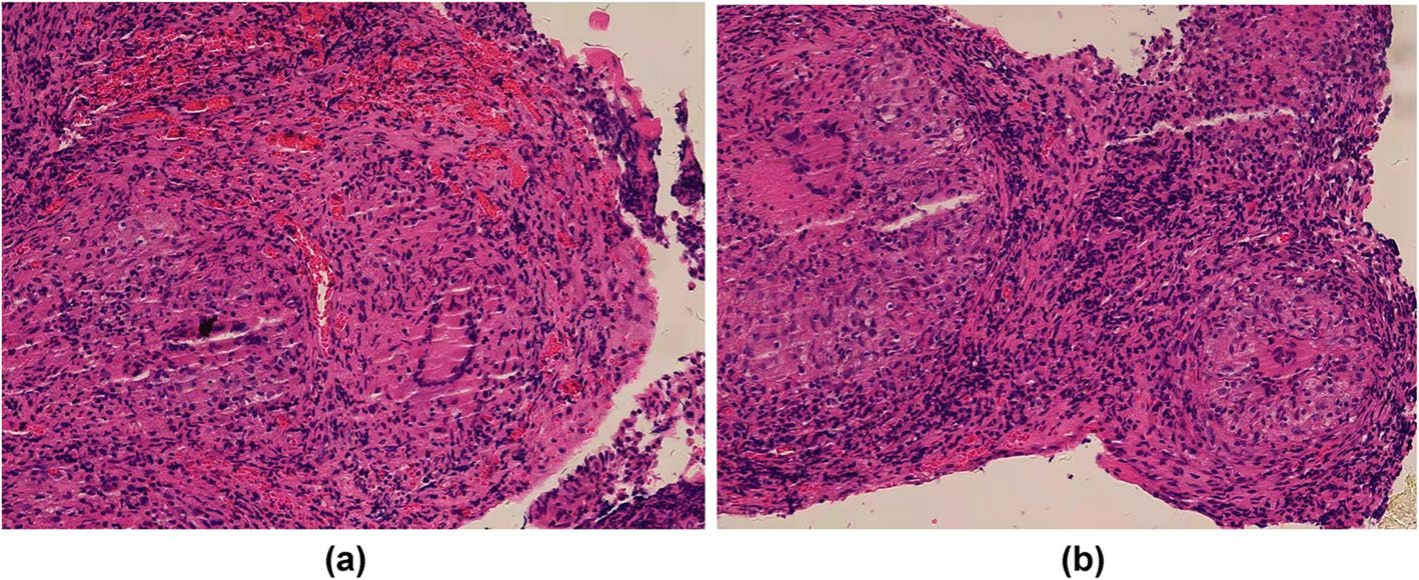
**a**, **b** Histopathology of pleural biopsy specimen showed granulomatous changes

At the same time, pleural effusion was sent for metagenomic next-generation sequencing (mNGS, mNGS refers to a high-throughput technology that identifies the nucleic acid sequences extracted from biological samples and yields information on the type and abundance of microorganisms contained in the sample [1]) and the result was unexpectedly negative. After expectant treatment for 2 weeks, there was no recurrence of cough, dyspnea, or fever. However, the patient still had the right pleural effusion with purulent yellow color 23 days after his admission (Fig. 2) suggesting empyema. The laboratory findings demonstrated ADA level in the right pleural effusion was increased (118.82 U/L) and the LDH level increased (7576.41 U/L) significantly. The right pleura was biopsied by CT-guided percutaneous needle and a pleural biopsy specimen was sent for mNGS and histopathology. Histopathology showed granulomatous changes (Fig. 3). The immunohistochemistry and special stains were negative. The presence of Mycobacterium avium complex (MAC) that only one sequence in the pleural tissue was finally identified on the 23rd day by mNGS.

**Fig. 4:**
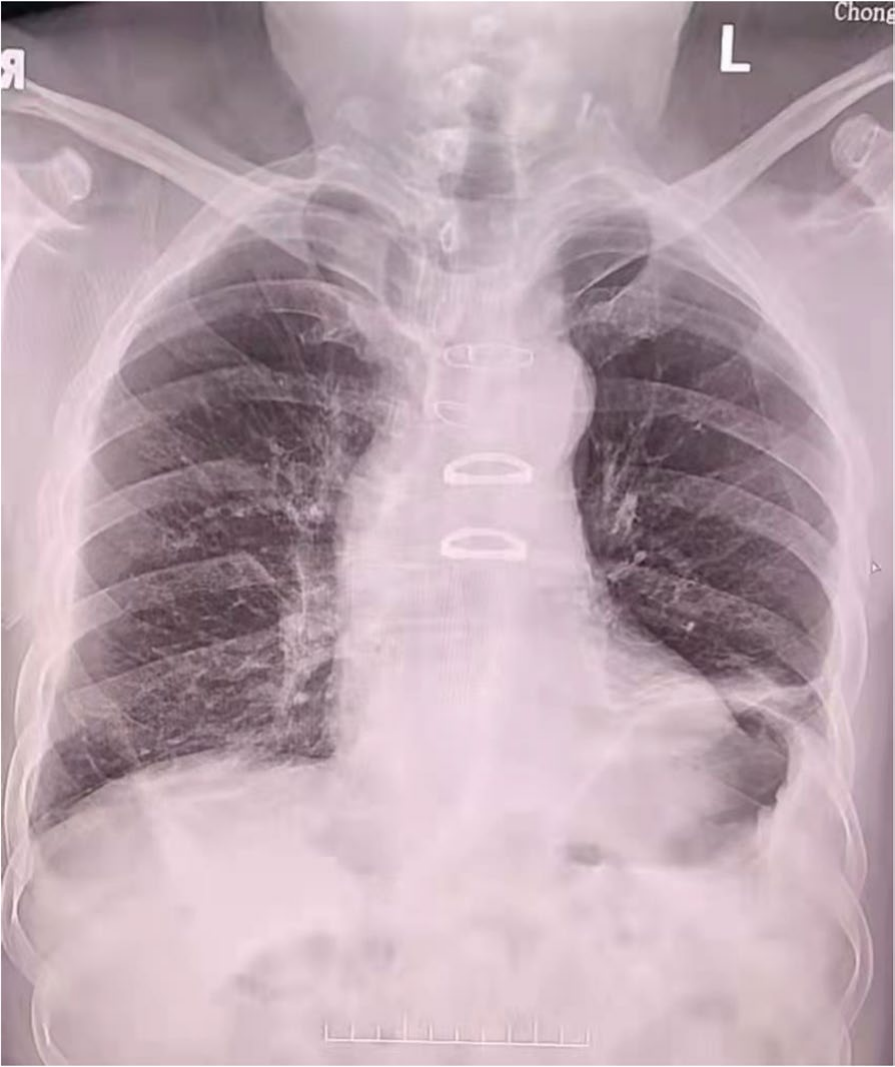
Chest x-ray performed at seven months after the start of antimicrobial treatment (March, 2022) showed the improvement of thoracic empyema

Based on the mNGS results, INH was switched to clarithromycin (CAM) (1.0 g/day) and amikacin(AK) (400 mg/day) in addition to RFP (450 mg/day) and EB (750 mg/day) began to treat the MAC infection according to national and domestic guidelines. The chest tube underwent continuous drainage. After expectant treatment for ten days, the ADA level in the right pleural effusion was decreased (86.22 U/L) and the LDH level decreased (4757.30 U/L). CT scan was taken about two weeks after treatment initiation against MAC (Fig. 1). When the flow of the right pleural effusion is less than 15 ml, the drainage tube was withdrawn. Chest x-ray performed about seven months later showed the improvement of thoracic empyema (Fig. 4).

## Discussion

(1) In recent years, NTM is increasingly recognized as a notifiable disease of both pulmonary and extrapulmonary sites, which remains challenging to diagnose and manage in China and globally [3, 5–7]. In addition, PNTM accounts for 90% of all nontuberculous mycobacterial infections [3, 8]. Pleural involvement or empyema by NTM is an extremely rare condition in patients who normally had a history of immunocompromised status or cancer. Moreover, NTM pleurisy has always been ignored because the laboratory characteristics of NTM pleuritis are similar to those of tuberculous (TB) pleuritis occasionally [3, 9]. Guidelines for the diagnosis of pleurisy caused by NTM have not been established, thus the diagnosis of NTM pleurisy, especially empyema, remains challenging [10]. In previous studies, the frequency of pleural effusion in patients with NTM infection was reported to be 1.4–6% [11–13]. In the reports of Sojung Park [14] and Takahiro Ando [11], patients with pleural effusion all had enrolled NTM lung disease. Moreover, in the past five years, no more than 10 cases of empyema by NTM were reported the worldwide. Therefore our case which is empyema by NTM neither pulmonary involvement nor immunodeficiency can improve our knowledge of the current situation of the NTM epidemic and allow us to build up a capacity for diagnosis and treatment at local and international levels [9].

(2) The 2020 ATS/IDSA guideline [15] and the Chinese NTM guideline both recommend the use of clinical, radiographic, and microbiologic criteria for diagnosing NTM pulmonary disease. The patients with NTM pleurisy are selected with the additional criteria (1) positive culture of NTM from pleural effusion or pleural biopsy or (2) positive smear of pleural effusion or pleural biopsy and positive result of nucleic acid amplification test for M. avium complex [11]. Here, the culture, specific probes and molecular biology techniques are recommended for diagnostic methods for NTM extrapulmonary disease, especially when it is the only manifestation without any pulmonary abnormities. In some cases, the definite diagnosis of NTM pleuritis will be made in patients who had NTM isolated. However, it is not clear if a single positive response of pleural effusion or pleural tissue for NTM in the proper context may be enough evidence for NTM pleurisy to initiate treatment because the same NTM species should be isolated in ≥ 2 sputum cultures which is the criteria in current guideline [15]. Moreover, as we knew, the clinical significance of NTM strains isolated from different clinical specimens is different [16]. The isolation of NTM from pleural effusion or pleural tissue often means pathogenic bacteria because it usually should exclude the possibility of environmental contamination. But we should treat cautiously samples that are isolated from respiratory tract specimens such as sputum and bronchoalveolar lavage fluid. Shu et al. reported that the prognosis of NTM pleurisy patients with single and multiple positive results were not markedly different [4, 17, 18].

Previous researches have emphasized the importance of early diagnosis and immediate therapy for progressing NTM pleurisy for this situation is associated with a high mortality rate which was reported to be 37–66% at 1 year for delayed diagnosis [17]. There are several clues associated with this diagnostic dilemma. Firstly, patients of pulmonary NTM with pleural effusion will probably develop NTM pleuritis simultaneously. NTM pleuritis commonly have a small amount of pleural effusion and NTM pleuritis can develop within 2 years after the diagnosis of pulmonary NTM. Secondly, the characteristics of pleural effusion are important threads. In previous reports, the characteristics of NTM-associated pleurisy are unknown in comparison to tuberculous pleurisy and the predominant cell types of pleural effusion were different [18, 19]. Most cases revealed a high leukocyte count with a low percentage of lymphocytes and elevated ADA levels in the pleural effusion. The clinical characteristics of the patient in our case report were similar to Takahiro’s research [11]. In our report, empyema effusion with high adenosine deaminase (ADA) activity in the pleural effusion which was increasing within 1 month was an important reminder, the ADA level of this patient roses from 36.67 to 118.82 U/L. In other reports, it was 152.7 U/L, ranging from 43.4 to 303 U/L [11]. In other reports, pleural effusion tests were similar to tuberculous pleurisy with a high percentage of lymphocytes and elevated ADA levels which still would remind you of the possibility of nontuberculous Mycobacterium infection [14]. Thirdly, several patients with NTM pleurisy had pneumothorax [20]. The NTM infection probably leaks from the pulmonary lesion to the thorax through perforating foci like pneumothorax. If a patient with pleural effusion/empyema and pneumothorax, physicians should consider the possibility of NTM but not tuberculosis. Fourthly, if the patient with pleural effusion does not respond to the initial treatment of anti-tuberculosis, physicians should think of the possibility of NTM except for bacterial infection or malignancy-related process.

(5) Many methods have been used for NTM diagnosis but mycobacterial culture and identification of NTM are not routinely performed in most areas in China. In our case, empyema was the only manifestation. Therefore, invasive methods such as closed thoracic drainage had been devised and pus was sent for TB-DNA, mNGS, TB and bacterial culture. Bacterial culture did not grow any organism and mNGS of pus was negative. Then Mycobacterial culture needs too long time that diagnosis was not made immediately. Finally, a pleural biopsy was chosen. The pathogen from pleural tissue was confirmed to be MAC by mNGS which is the means with the highest resolution for species identification. As already stated, consideration of nontuberculous empyema is very difficult because of the low diagnostic yield. Furthermore, negative results of laboratory examination explain the dilemma in diagnosing NTM in this case. In our experience, despite the negative laboratory analysis demonstrated, a further pleural biopsy was performed and showed a decisive diagnostic tool for NTM pleuritis [14].

## Conclusion

In our immunocompetent patients, NTM empyema without pulmonary disease should be considered as a diagnostic dilemma. The pleural fluid should be tested for mycobacteria on culture or PCR or mNGS so that identification of species can be done and treatment can be tailored accordingly. Therefore, physicians should take particular care in diagnosing NTM pleurisy.

## Data Availability

The datasets used and/or analysed during the current study are available from the corresponding author on reasonable request.

## Abbreviations

NTM: Non-tuberculous mycobacteria
ADA: Adenosine deaminase
mNGS: Metagenomic next-generation sequencing
MAC: M. avium-intracellulare complex
CRP: C-reactive protein
PCT: Procalcitonin
CT: Computed tomography
LDH: Lactic acid dehydrogenase
PCR: Polymerase chain reaction
IGRA: Interferon-gamma release assay
INH: Isoniazid
RFP: Rifampicin
EB: Ethambutol
AK: Amikacin
PNTM: Pulmonary non-tuberculous mycobacteria
TB: Tuberculous
TB-DNA: Tuberculous-deoxyribonucleic acid
