# Correction: Thoracic empyema due to nontuberculous mycobacteria in an immunocompetent patient without pulmonary disease: a case report

**DOI:** 10.1186/s12890-023-02564-8

**Published:** 2023-07-19

**Authors:** Fengjiao Yu, Yongxia Li, Jing Luo, Xingru Chen, Yu Jiang

**Affiliations:** Department of Respiratory and Critical Care Medicine, University-Town Hospital of Chongqing Medical University, Chongqing, 401331 People’s Republic of China

**BMC Pulmonary Medicine (2023) 23:215**

10.1186/s12890-023-02494-5

Following publication of the original article [1], it came to the authors’ attention that the given and family name of the corresponding author had been erroneously swapped. The name has since been corrected (to Yu Jiang) and the corrected name may be seen in the author list of this erratum.

The authors thank you for reading this erratum and apologize for any inconvenience caused.

